# Structured, Harmonized, and Interoperable Integration of Clinical Routine Data to Compute Heart Failure Risk Scores

**DOI:** 10.3390/life12050749

**Published:** 2022-05-18

**Authors:** Kim K. Sommer, Ali Amr, Udo Bavendiek, Felix Beierle, Peter Brunecker, Henning Dathe, Jürgen Eils, Maximilian Ertl, Georg Fette, Matthias Gietzelt, Bettina Heidecker, Kristian Hellenkamp, Peter Heuschmann, Jennifer D. E. Hoos, Tibor Kesztyüs, Fabian Kerwagen, Aljoscha Kindermann, Dagmar Krefting, Ulf Landmesser, Michael Marschollek, Benjamin Meder, Angela Merzweiler, Fabian Prasser, Rüdiger Pryss, Jendrik Richter, Philipp Schneider, Stefan Störk, Christoph Dieterich

**Affiliations:** 1 Peter L. Reichertz Institute for Medical Informatics, TU Braunschweig and Hannover Medical School, Carl-Neuberg-Straße 1, 30625 Hannover, Germany; sommer.kimkatrin@mh-hannover.de (K.K.S.); gietzelt.matthias@mh-hannover.de (M.G.); marschollek.michael@mh-hannover.de (M.M.); 2 Department of Internal Medicine III (Cardiology, Angiology, and Pneumology), University Hospital Heidelberg, Im Neuenheimer Feld 410, 69120 Heidelberg, Germany; ali.amr@med.uni-heidelberg.de (A.A.); a-kindermann@gmx.net (A.K.); benjamin.meder@med.uni-heidelberg.de (B.M.); philipp.schneider@uni-heidelberg.de (P.S.); 3 German Center of Cardiovascular Research (DZHK), Partner Site Heidelberg/Mannheim, 69120 Heidelberg, Germany; 4 Department of Cardiology and Angiology, Hannover Medical School, Carl-Neuberg-Straße, 130625 Hannover, Germany; bavendiek.udo@mh-hannover.de; 5 Institute of Clinical Epidemiology and Biometry, University of Würzburg, Am Schwarzenberg 15, 97078 Würzburg, Germany; felix.beierle@uni-wuerzburg.de (F.B.); peter.heuschmann@uni-wuerzburg.de (P.H.); pryss_r@ukw.de (R.P.); 6 Core Facility IT, Berlin Institute of Health at Charité—Universitätsmedizin Berlin, Charitéplatz 1, 10117 Berlin, Germany; peter.brunecker@charite.de (P.B.); jennifer.hoos@charite.de (J.D.E.H.); 7 Medizinisches Datenintegrationszentrum, Charité—Universitätsmedizin Berlin, Charitéplatz 1, 10117 Berlin, Germany; 8 Department of Medical Informatics, University Medical Center Göttingen, Robert-Koch-Str. 40, 37075 Göttingen, Germany; henning.dathe@med.uni-goettingen.de (H.D.); tibor.kesztyues@med.uni-goettingen.de (T.K.); dagmar.krefting@med.uni-goettingen.de (D.K.); jendrik.richter@med.uni-goettingen.de (J.R.); 9 Center Digital Health, Berlin Institute of Health at Charité—Universitätsmedizin Berlin, Charitéplatz 1, 10117 Berlin, Germany; juergen.eils@charite.de; 10 Datenintegrationszentrum (DIZ), Servicezentrum Informatik (SMI), Universitätsklinikum Würzburg (UKW), Schweinfurter Strasse 4, 97078 Würzburg, Germany; ertl_m@ukw.de (M.E.); georg.fette@uni-wuerzburg.de (G.F.); 11 Medizinische Klinik für Kardiologie, Charité—Universitätsmedizin Berlin, Charitéplatz 1, 10117 Berlin, Germany; bettina.heidecker@charite.de (B.H.); ulf.landmesser@charite.de (U.L.); 12 Department of Cardiology and Pneumology/Heart Center, University Medical Center Göttingen, Robert-Koch-Str. 40, 37075 Göttingen, Germany; kristian.hellenkamp@med.uni-goettingen.de; 13 Department Klinische Forschung und Epidemiologie, Deutsches Zentrum für Herzinsuffizienz, Am Schwarzenberg 15, 97078 Würzburg, Germany; kerwagen_f@ukw.de (F.K.); stoerk_s@ukw.de (S.S.); 14 Medizinische Klinik I, Universitätsklinikum Würzburg (UKW), Oberdürrbacherstraße 6, 97080 Würzburg, Germany; 15 German Center of Cardiovascular Research (DZHK), Partner Site Göttingen, Robert-Koch-Straße 42a, 37075 Göttingen, Germany; 16 Institute for Cardiomyopathies Heidelberg (ICH), Heidelberg University Hospital, Im Neuenheimer Feld 669, 69120 Heidelberg, Germany; 17 Institute of Medical Informatics, Heidelberg University Hospital, Im Neuenheimer Feld 130.3, 69120 Heidelberg, Germany; angela.merzweiler@med.uni-heidelberg.de; 18 Medical Informatics Group, Berlin Institute of Health at Charité—Universitätsmedizin Berlin, Charitéplatz 1, 10117 Berlin, Germany; fabian.prasser@bih-charite.de; 19 Klaus Tschira Institute for Integrative Computational Cardiology, Heidelberg University Hospital, Im Neuenheimer Feld 669, 69120 Heidelberg, Germany

**Keywords:** medical informatics initiative, HiGHmed, medical data integration center, clinical routine data, heart failure, risk prediction scores, semantic interoperability, openEHR

## Abstract

Risk prediction in patients with heart failure (HF) is essential to improve the tailoring of preventive, diagnostic, and therapeutic strategies for the individual patient, and effectively use health care resources. Risk scores derived from controlled clinical studies can be used to calculate the risk of mortality and HF hospitalizations. However, these scores are poorly implemented into routine care, predominantly because their calculation requires considerable efforts in practice and necessary data often are not available in an interoperable format. In this work, we demonstrate the feasibility of a multi-site solution to derive and calculate two exemplary HF scores from clinical routine data (MAGGIC score with six continuous and eight categorical variables; Barcelona Bio-HF score with five continuous and six categorical variables). Within HiGHmed, a German Medical Informatics Initiative consortium, we implemented an interoperable solution, collecting a harmonized HF-phenotypic core data set (CDS) within the openEHR framework. Our approach minimizes the need for manual data entry by automatically retrieving data from primary systems. We show, across five participating medical centers, that the implemented structures to execute dedicated data queries, followed by harmonized data processing and score calculation, work well in practice. In summary, we demonstrated the feasibility of clinical routine data usage across multiple partner sites to compute HF risk scores. This solution can be extended to a large spectrum of applications in clinical care.

## 1. Introduction

Chronic heart failure is a frequent condition of the elderly and has a population prevalence of 2–6% [1,2]. In Germany, heart failure affects more than two million people and is the most frequent reason for hospitalization [3,4]. The causes and manifestations of heart failure syndrome are manifold, with comorbidities complicating the disease trajectory. The typical course of progression shows a fluctuating pattern with frequent episodes of de- and recompensation [2], which associate with repeated hospitalizations and death and have a major impact on patients’ quality of life. Thus, predictive modelling for an individual patient—and its application in clinical routine, e.g., in the frame of a score—is key in heart failure care, as it allows improved tailoring of preventive [5], diagnostic and therapeutic measures [6]. Despite their abundant availability [7], the uptake and implementation of heart failure risk scores in clinical practice has been slow, likely due to major barriers. These include limited or lacking availability of score-relevant information in structured clinical records, insufficiently standardized and/or incompletely stored information in electronic health records (EHRs), and time-consuming manual compilation and/or computation procedures. The advances seen in the areas of medical informatics, high-performance cluster computing, and big data processing hold promise to also alleviate score derivation and to augment the clinical use of scoring schemes.

The HiGHmed Consortium, funded by the German Ministry of Education and Research (BMBF), in the context of the German Medical Informatics Initiative, comprises eight University Medical Centers and several academic and industrial partners. HiGHmed aims to enable the sharing of health data from clinical routine and analytics tools for research and clinical care [8,9]. HiGHmed has developed and implemented an open platform approach to achieve syntactic and semantic interoperability designed for open information models, open application programming interfaces, and open service models. This, in turn, is expected to result in improved, future-oriented patient care. HiGHmed bundles and integrates the core competencies of its partners, establishing local medical data integration centers (MeDICs) and they are working together across organizations and institutions on novel, interoperable solutions in medical informatics. The research work described here was conducted within the HiGHmed Use Case Cardiology (UCC). There, clinicians, medical informaticians, and data scientists, from seven University Hospitals (Berlin, Cottbus, Göttingen, Hannover, Heidelberg, Kiel, and Würzburg) and numerous partners from science, industry, and healthcare joined forces to improve the long-term healthcare of patients with chronic heart failure, using medical informatics methods. All sites involved in the UCC agreed on standardized data models as a basis for exchanging and curating data for research. As a basis, they use the data model of the German Center for Cardiovascular Research e.V. (DZHK, https://dzhk.de, accessed on 1 March 2022). Uniformly collected and documented information at respective points of care is extracted in a pseudonymized fashion, aggregated in MeDICs, and made available for joint analyses.

The data model and interoperability approach of the UCC are implemented in openEHR, which describes the management, storage, retrieval, and exchange of health data in EHRs (https://www.openehr.org, accessed on 1 March 2022). All MeDICs host local openEHR platforms, which collect all required data items for data sharing and analysis. Two main aspects represent the formalization of clinical data in openEHR models: (1) Archetypes represent the definition of a clinical concept (e.g., blood pressure measurement, including systolic and diastolic value and associated metadata); (2) Templates combine multiple archetypes to represent a defined clinical situation (e.g., medical history, echocardiography, etc.). The UCC currently employs 51 archetypes in 12 different, templates resulting in more than 350 data items that were either newly created or re-used from internationally available or HiGHmed native models (see https://ckm.highmed.org/, accessed on 1 March 2022). The data collection approach aimed to minimize the need for manual data entry by automatically retrieving data from primary systems via extract, transform, and load processes (ETL).

We aimed to demonstrate the feasibility and utility of a multi-site solution, allowing to derive two selected heart failure scores: the MAGGIC score [10], which comprises five continuous and eight categorical variables, and the Barcelona BioHF score v1 [11], which comprises five continuous and six categorical variables. Both scores have been validated previously to predict one-year and three-year mortality, respectively. Table 1 summarizes the respective components of each score.

## 2. Materials and Methods

### 2.1. Management

The UCC group consisted of cardiologists and medical informatics experts, with at least one representative from each participating HiGHmed site attending weekly web conferences. This diversity of expertise was essential to meet the goal of implementing data models for the purpose of calculating heart failure scores from routine clinical data. In addition to web conferences, GitLab was used for project management, i.e., to assign tasks and to monitor project progress.

### 2.2. Data

The UCC conducts an ongoing observational multi-site prospective cohort study. To date, more than 3500 patients agreed to provide their data from medical history and clinical examinations at the participating sites, as well as respective information collected during bi-annual follow-up visits.

The following inclusion criteria apply for the HiGHmed cohort:

(1) Age ≥ 18 years;
(2) Established diagnosis of chronic heart failure;
(3) Capacity to consent;
(4) Completed patient information and written informed consent.

The following exclusion criteria were defined:

(1) Life expectancy less than 6 months due to non-cardiac pre-existing conditions;
(2) Incapacity to consent.

Patient recruitment started after approval of the study protocol in 2018 and was planned for 4 years. Berlin and Würzburg joined the project two years later.

The selection of study participants was left to the sites and therefore is very heterogeneous. In Heidelberg, for example, only outpatients were recruited; in Hannover, mainly inpatients.

### 2.3. OpenEHR Archetypes and Templates

For clinical data storage all sites host the openEHR-based Better platform (https://www.better.care, accessed on 24 February 2022)), whereas Göttingen used the open source solution EHRbase (https://github.com/ehrbase/ehrbase, accessed on 24 February 2022) to assess cross-vendor interoperability. We used the following openEHR templates for score calculation: Medical History, Medication, Echocardiography, Laboratory, Personal Data, and Study Participation. The templates and data items are jointly listed in Table 1. We additionally recorded information on whether a patient was admitted as an outpatient or inpatient at the time of study inclusion as additional covariate.

### 2.4. Primary Systems and Data Integration

Site-specific infrastructures and prerequisites determine the primary systems from which clinical data are extracted for integration into the openEHR platform. Table 2 lists the primary systems from which the data for the score calculation originated per site and template. For some templates, data were specifically recorded by UCC study nurses; for other templates, the data were taken from routine care. The data integration processes varied highly across the sites, and the description of the process for the exact steps that were performed to integrate the data into the openEHR platform is beyond the scope of this manuscript.

### 2.5. Data Retrieval and Preparation

For data retrieval and preparation, we used a script written in the programming language R, which was managed and shared across the sites via GitLab. The code was executed independently by each site on their local data store. We ensured a consistent software environment by using R library renv version 0.15.1. The sites Hannover, Würzburg and Berlin directly queried the openEHR platform using the archetype query language (AQL) through REST directly from the R script, Heidelberg executed the semantically same AQL queries in Better EHR Studio 2.3.0 (MeDIC) and transferred the results in CSV format to the local data analysts. Göttingen used slightly adapted AQL queries due to subtle incompatibilities between EHRbase 0.19.0 and the Better platform. All queries are restricted to patients enrolled in the UCC by appending the patient identifiers to the query. The executed queries can be found in the Appendix A (Query A1–A7).

The subsequent processing steps in R (HIGHmedUCCScores) were the same for all sites and comprised the following:

- Personal data: calculate age from birth date.
- Medical history: calculate BMI from weight and height, calculate HF duration and HF duration ≥ 18 months, assign levels to categorical variables.
- Medication: (1) we use information on medication that is closest to the date of patient recruitment. (2) If applicable, we map medication groups from the openEHR template to synonymous groups required for score calculation.
- Echocardiography: again, we use the information closest to the date on which the patient was recruited.
- Laboratory Data: again, we use the information closest to the date on which the patient was recruited.

Subsequently, the 1-year mortality and the 3-year mortality for the Barcelona BioHF V1 Score and the MAGGIC Score were calculated using the HiGHmedUCCScore package (https://github.com/dieterich-lab/HiGHmedUCCScores, accessed on 23 February 2022). Barcelona BioHF V1 Score also computes 2-year mortality, but MAGGIC score does not, so we opted for predicting 1 and 3-year mortality only. All patient identifiers were replaced with pseudonyms (Patient G1, H2, etc.) before sharing clinical information with the other sites.

### 2.6. Plausibility Checks

Both the BioHF1 and the MAGGIC Score enforce upper and lower boundaries on numerical values. Any values outside are set to the upper or lower boundary, respectively. However, implausible values (e.g., incorrect record entries) are simply not detected by this mechanism and are automatically set. For this reason, we implemented additional plausibility checks before score calculation. If any given value falls outside the defined limits (see Table 3), we consider it a missing value and report the respective record at execution time.

### 2.7. Score Calculation

Following data retrieval, processing and quality control, both scores are computed by the HiGHmedUCCScores package based on the original publications [10,11]. The R package allows one to predict the 1-year mortality as well as the 3-year mortality for both scores. As described by the authors of the scores, imputation of missing values is possible for the Barcelona BioHF V1 score, but not for the MAGGIC score. In summary, both scores provide prognostic information on future patient status [10,11].

### 2.8. Further Assumptions

Owing to the fact that the dataset was not designed in advance to calculate the HF scores, but was intended for use in routine care, we made the following assumptions in variable mapping:

- Personal data—birth date: Both scores require the age of the patient. Due to privacy restrictions, the year of birth, but not the exact birth date, was oftentimes only available for many patients. In the absence of an exact birth date, we set the birth date to the 1st July of the respective year.
- Medical history—COPD: The MAGGIC score requires the diagnosis of COPD, which is by definition based on a spirometry measurement [12]. This was replaced with information extracted from anamnesis.
- Patient history—first diagnosis date of HF: The Barcelona BioHF V1 and the MAGGIC score require the HF duration and the HF duration ≥ 18 months on monthly precision, respectively. For patients who had only the year of the first diagnosis of HF documented, we set the date of HF onset to the 1st July of the respective year.
- Medication: Information that a drug was not prescribed is not documented in routine clinical practice. If the drug was not recorded in the medication list closest to the medical history date, it was assumed that it was not taken.
- Medication—loop diuretic: The Barcelona BioHF Score requires dosing information in terms of furosemide and torasemide equivalents. In case of absence of this information, we equated documented loop diuretic use as the lower dosage of furosemide, because this was the usual dosage at all participating hospitals.

## 3. Results

### 3.1. The OpenEHR Approach, Data Queries, Processing, and Score Calculation Steps

All partner sites continued to apply their proprietary method for data documentation and integration. Yet, given the heterogenic IT systems landscape, individual ETL concepts had to be established. Nevertheless, all sites were able to store data in the required templates in their local openEHR platform for recruited HiGHmed patients. Evidently, the data integration processes varied highly across the different partner sites, and individual documentation and ETL concepts had to be established.

By using consistent archetypes and templates in openEHR, it was possible to run our queries at the five partner sites. Queries are listed in Appendix A. We performed all queries per template as opposed to per patient, as this approach requires far fewer queries to the platform. However, we noticed that queries should be split into defined batch sizes (e.g., 400 patients) due to size and time limitations on single queries. Unexpectedly, we noticed subtle incompatibilities between the open source EHRbase solution (0.19.0, used by Göttingen) and the commercial Better EHR Platform. For example, the Better platform accepts different permutations of LIMIT and OFFSET, e.g., “OFFSET LIMIT” instead of “LIMIT OFFSET”. However, the specification (https://specifications.openehr.org/releases/QUERY/latest/AQL.html#_limit, accessed on 1 March 2022) states that LIMIT should precede OFFSET.https://specifications.openehr.org/releases/QUERY/latest/AQL.html#_limit, accessed on 1 March 2022) states that LIMIT should precede OFFSET.https://specifications.openehr.org/releases/QUERY/latest/AQL.html#_limit, accessed on 1 March 2022) states that LIMIT should precede OFFSET.https://specifications.openehr.org/releases/QUERY/latest/AQL.html#_limit, accessed on 1 March 2022) states that LIMIT should precede OFFSET.

Since the results of the queries were in the same format at all sites, the steps for calculating the score were identically performed. Thus, all final data and computed scores were available in the same format at all sites, making it possible to merge the data sets without effort after sharing. The original data, thus, remained at the individual sites at all times and only the completely anonymized data set was shared.

### 3.2. Data Integration

**Figure 1:**
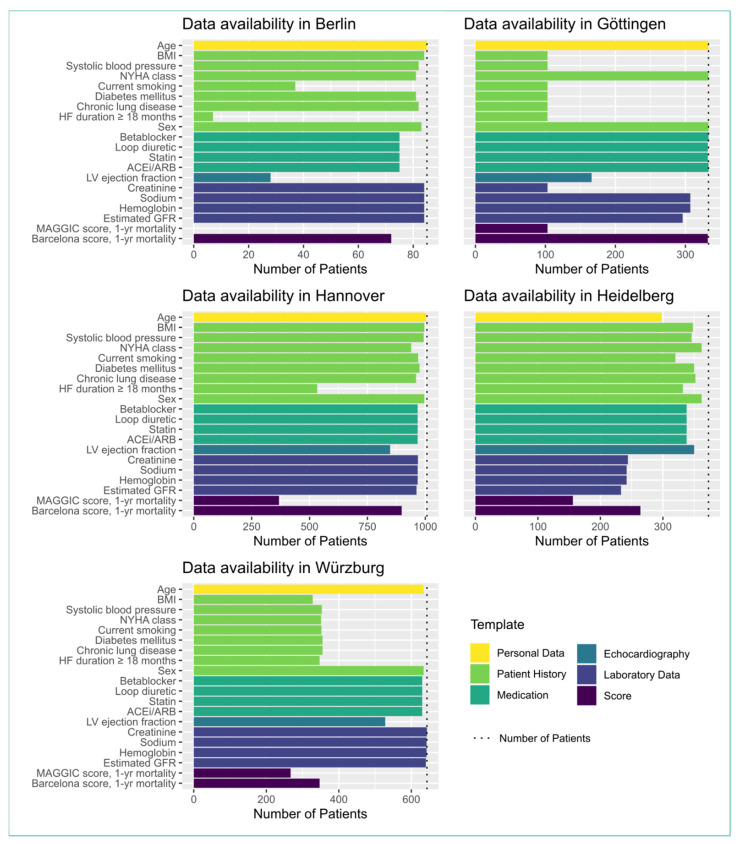
Number of available data items for HF score calculation at each of the five participating partner sites. Entities over five openEHR template classes are shown (see color code) and the number of successfully computed HF scores. The upper boundary (i.e., the number of considered individual patients) is shown as dashed line.

Figure 1 shows bar charts depicting the amounts of available data items in the openEHR repositories at the different clinical sites. The charts are grouped by color-coded template type. Additionally, there are two bars showing the number of patients for which all necessary data items were present to compute the two scores. The availability of routine data sources varied across the clinical sites. The reasons for reduced availability of the data sources were manifold:

1. If a necessary data type was rarely collected during routine visits or was not collected at all, these data were lacking for the study. In Berlin, the HF duration ≥ 18 months and the smoking status were taken from discharge letters, which seldom contained this information or the information was asked from the patients themselves, who often could not properly remember the exact point in time of their first heart failure event. Therefore, those data types had a low availability in Berlin.
2. At some sites, the collection of data within some source systems began after the start of the study. This is the case for Würzburg, where the patient history was collected via a dedicated patient history form. This form was implemented after the start of the study. Therefore, the patient history data were only available for patients for which the anamnesis data were collected using this form, or for returning patients who visited the clinic another time.
3. We tried to automate the transfer to the target openEHR systems as much as possible. However, due to technical or organizational reasons, there were parts of the ETL processes where manual work was needed to integrate the data into the target systems. In Berlin, the echocardiography report was transcribed manually from the source system to the openEHR template. This led to a reduced availability in the LVEF values in Berlin.
4. In an ideal scenario, ETL processes directly retrieve data from streams originating from the source systems. This ensures that the target system (i.e., MeDIC) is in sync with the source system (e.g., by listening to an HL7-communication server). Unfortunately, some partner sites use data snapshots rather than data streams to fill their openEHR platform. This may lead to very different proportions of recruited vs. documented patients at the respective partner sites.

The MAGGIC score relies on complete data. Therefore, the number of patients with a computed MAGGIC score is bounded by the least available data item (see Figure 1). Still, the number of scores can be even lower, because the available data sets for all patients are not completely overlapping, i.e., some patients are missing one data item, whereas others are missing something else. In some extreme cases, this may lead to partner sites reporting a very low number of MAGGIC scores in comparison to the number of documented patients.

For four of the sites, the HF duration ≥ 18 months data item was the limiting factor for calculating the MAGICC score. The HF-duration data was either requested from the patients themselves within a patient history form (Würzburg, Berlin, Hannover) or calculated from historic diagnostic data, from either accounting data (i.e., ICD10-encoded billing data) (Würzburg) or from historic patient discharge letters (Berlin). In Heidelberg, the amount of MAGGIC scores is far lower than even the availability of HF-duration data because other data items were missing.

### 3.3. Statistical Analysis

The MAGGIC score utilizes clinical and medication data (see Table 1). Taken together, we could compute MAGGIC scores for 894 patients out of 2441 recruited patients. The BioHFv1 score uses a different feature set (see Table 1), enabling us to calculate the score values of 1899 patients and 1352 patients with imputation and without imputation, respectively.

**Figure 2:**
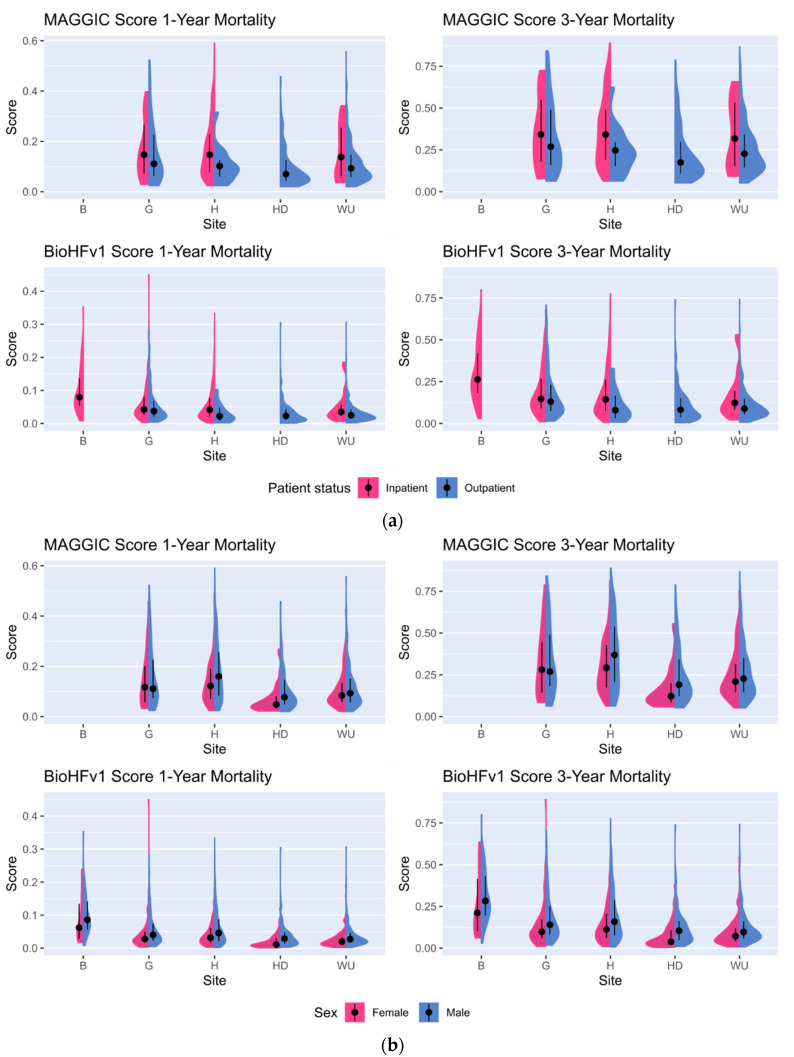
The split violin plots for the MAGGIC score and the BCN BioHFv1 score (1- and 3-year mortality risk, respectively) across the different clinical sites. Black dots show median values and thin black lines show upper and lower quartiles. We either stratified by (**a**) patient status (inpatient vs. outpatient) or by (**b**) patient sex (female vs. male). Partner site codes: B = Berlin, G = Göttingen, H = Hannover, HD = Heidelberg, WU = Würzburg.

Figure 2a shows that all median score values are lower for outpatients as compared to inpatients for all participating sites where both patient groups were recruited. Figure 2b indicates that females tend to have a lower mortality risk as compared to males. Evidently, sex is one relevant feature in score computation. For example, males receive a higher score in MAGGIC (+1) than females. This is the easiest explanation for the observed pattern. However, in Göttingen, the median MAGGIC score for females is slightly above the median score for males.

For the MAGGIC score, Table 4 stratifies these results by inpatients vs. outpatients.

Table 4 already indicates a heterogeneous patient population across partner sites. A Kruskal–Wallis test over all patients rejects the null hypothesis of similar MAGGIC scores for all partner sites (*p*-value < 1.49 × 10^−15^). If we restrict the same test to outpatients only, the *p*-value increases to ~6 × 10^−^⁴, which hints at patient status being one of the key factors in determining score differences.

**Figure 3:**
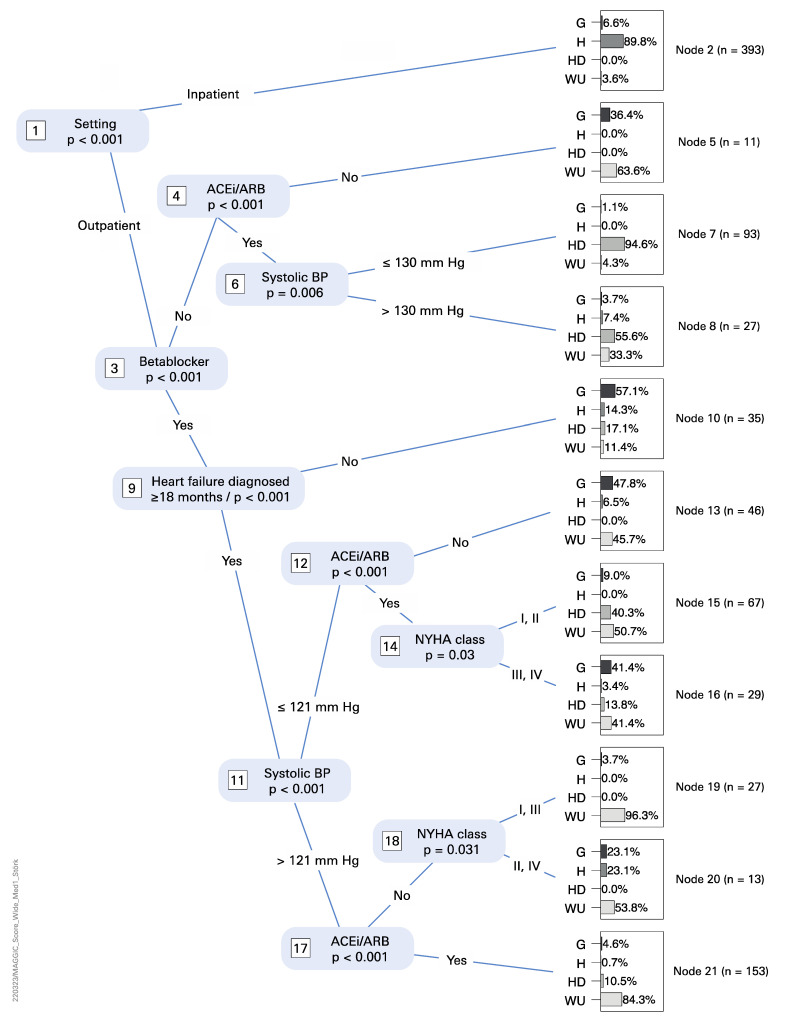
Conditional inference tree for MAGGIC score features (see Table 1). Score features are used to assign patients to partner sites and to pinpoint differences in the site-specific patient cohorts. Terminal nodes show patient proportions over sites and the total number of patients, respectively.

We further corroborated our analysis using a conditional inference tree approach [13] to uncover MAGGIC score features that might explain these differences across four partner sites. Briefly, all 14 features that are used to compute the MAGGIC score were used to predict the origin of a given patient. Figure 3 shows that six features are informative to predict patient origin (i.e., differ between partner sites): patient status, use of beta blockers and ACE inhibitors, systolic blood pressure, NYHA classification, and first diagnosis date of HF. For example, node 7 in Figure 3 shows that a subset of Heidelberg patients is characterized by outpatient status, absence of beta blockers, presence of ACE inhibitors, and a systolic blood pressure of ≤130 (i.e., absence of hypertension).

The BioHFv1 offers the possibility to impute missing values and, thus, could cover a larger patient cohort (see Table 5).

Table 5 indicates a heterogenous patient population across partner sites. A Kruskal–Wallis test over patients with complete data rejects the null hypothesis of similar BioHFv1 scores for all partner sites (*p*-value < 1.73 × 10^−19^). If we restrict the same test to outpatients only, the *p*-value increases to ~0.0005, which hints at patient status being of one of the key factors here as well.

Some partner sites have more imputed BioHFv1 scores than computed from complete data. We tested independently for each partner site if BioHFv1 scores are significantly different. This is, indeed, the case. For example, a Wilcoxon test for Göttingen patients comparing imputed and complete BioHFv1 scores shows that imputed scores are significantly higher than non-imputed scores (*p*-value: ~3 × 10^−3^.). Similar observations have been made for other sites with sufficient numbers, such as Heidelberg and Hannover, but not for Würzburg patients (i.e., no significant differences, see also section on data integration/missing data).

**Figure 4:**
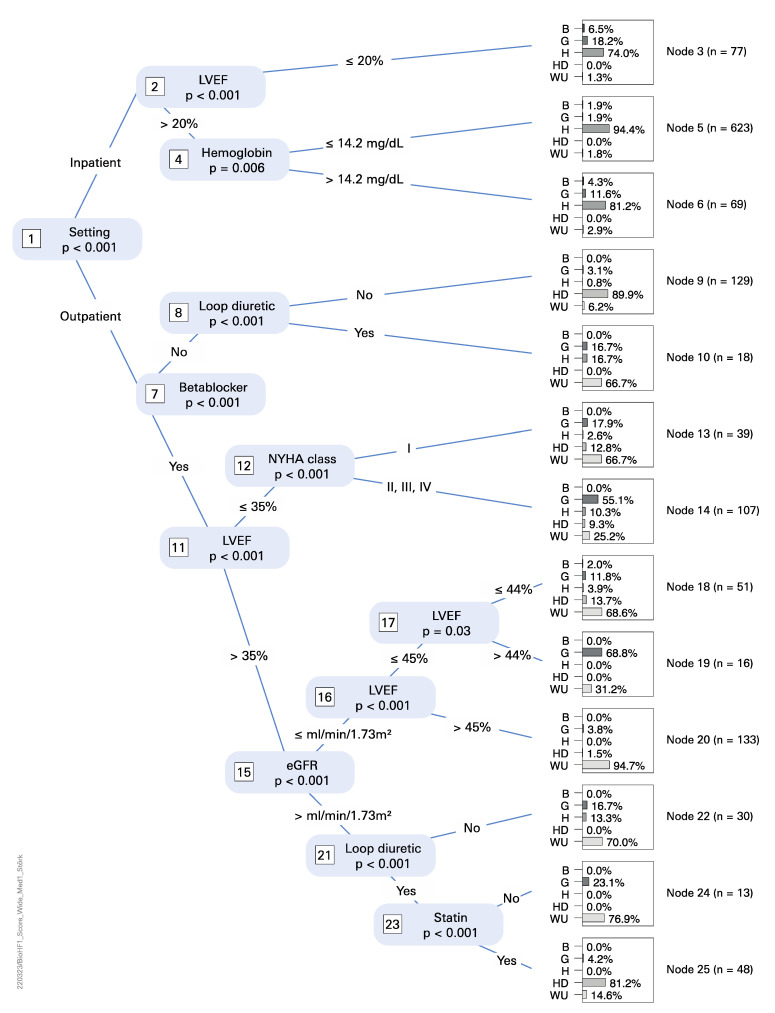
Conditional inference tree for BioHFv1 score features (see Table 1). Score features are used to assign patients to partner sites and to pinpoint differences in the site-specific patient cohorts. Terminal nodes show patient proportions over sites and the total number of patients, respectively.

We further corroborated our analysis using a conditional inference tree approach [13] to uncover BioHFv1 score features that might explain these differences across four partner sites. Briefly, all 14 features that are used to compute the MAGGIC score were used to predict the origin of a given patient. Figure 4 shows that eight features are informative to predict patient origin (i.e., differ between partner sites): patient status, beta blocker, LV ejection fraction, loop diuretic, Hemoglobin, NYHA class, estimated GFR and Statin. For example, terminal node 5 in Figure 4 shows that a subset of Hannover patients is characterized by inpatient status, an LVEF > 20, and Hemoglobin ≤ 14.2. This is hardly found anywhere else (i.e., Berlin, Göttingen, Heidelberg, Hannover). Another example is terminal node 20, where a specific subset of Würzburg patients was identified; namely, those outpatients who are treated with beta blockers have an LVEF of > 45 and an estimated GFR of less than ≤76.

## 4. Discussion

Risk prediction in patients with heart failure (HF) is essential to optimally tailor diagnostic and therapeutic strategies, and effectively use healthcare resources. Risk scores derived from controlled clinical studies can be used to calculate the risk of mortality and HF hospitalizations.

However, these scores are poorly implemented into routine care, predominantly because their calculation requires considerable efforts in practice. The HiGHmed Use Case Cardiology set out to demonstrate the feasibility of such an endeavor in a clinical routine setting. Five university hospitals teamed up to develop structured, harmonized, and interoperable documentation to compute two selected heart failure risk scores.

### 4.1. The OpenEHR Approach, Data Queries, Processing, and Score Calculation Steps

Although each of the five clinics had different requirements, we managed to develop a routine for data retrieval and score calculation that works well in practice at all sites. A major advantage was that data in the openEHR repository can be queried via the REST API, which can easily be implemented in various software solutions, including the widely used R environment. In summary, we developed a complete end-to-end workflow in R, from data retrieval to score calculation.

Moreover, the use of the same AQL queries at four out of five sites shows the great potential offered by semantic models and interoperable technologies. On the one hand, the data will be more comparable and can be used more easily for future multicenter studies. On the other hand, applications based on these data are interoperable, interchangeable and, therefore, easily implemented to all sites using the same technology. This reduces development costs significantly. However, we encountered a few compatibility issues for the Better platform vs. the EHRbase platform.

Specifically, the Better platform currently tolerates extensions to the AQL syntax that differ marginally from the openEHR specification. We see the need for discussing either a stricter implementation or modifications of the standard towards more error-tolerant interpretations of the queries to maintain interoperability. This discussion is already happening to some degree in the openEHR community.

### 4.2. Data Integration

The first apparent problem with data from clinical routine is missing data. As described in Section 3.2, there are two main reasons for missing data: (1) non-existence of data or (2) its non-availability. Figure 1 shows that the very problem of missing data exists at all the participating sites. At all sites, more Barcelona scores (algorithm allows imputation) could be calculated than MAGGIC scores (algorithm requires complete data). Therefore, we found that, for routine clinical practice, scoring algorithms that can handle missing values are preferable.

There are multiple methods for the resolution of the missing data problem, depending on the particular problem:

1. In the case of existing data, availability issues need to be solved, such as:
  (a) More ETL routes have to be established from the primary systems to the data integration centers.
  (b) Vendors of medical devices need to be encouraged to enable reusability of recorded data by integrating export APIs into their products.
  (c) Source systems, which currently provide data in unstructured free text form, have to be transformed into structured data, providing systems, so that no error-prone manual or automatic information extraction is needed.
2. In the case of missing data (e.g., the “HF duration ≥ 18 month” in the present study), reasons for its absence need to be identified. Potential reasons may include:
  (a) Time constraints on detailed documentation in routine care, e.g., it is too time consuming for the documenting physician to capture the data item.
  (b) Alternatively, the physician considers data as not necessary for a particular patient, despite its value in secondary use (e.g., research).

Along these lines, Table 2 shows the need to create several custom data collection methods at the different sites to mitigate the aforementioned problems for our study setting. To some extent, the core data set initiative (https://www.medizininformatik-initiative.de/en/medical-informatics-initiatives-core-data-set, accessed on 1 March 2022) of the medical informatics consortia will ensure that a minimal set of structured information is available across Germany (https://www.medizininformatik-initiative.de/en/medical-informatics-initiatives-core-data-set, accessed on 1 March 2022) of the medical informatics consortia will ensure that a minimal set of structured information is available across Germany.

A second problem, which is not in the focus of the present study, is the comparability of routine data for secondary use (e.g., clinical research). Clearly, source systems differed between all participating sites (see Table 2), which raises the question if the corresponding data could be seen as equivalent for a specific secondary use task (e.g., risk score computation).

In contrast to standard clinical studies where the data collection process is described in detail in study protocols, the quality of the results of studies relying on routine care data reuse always needs to be examined in the context of source-data specifics. Our approach should be seen as a support for standard clinical studies. Conventional studies can, e.g., be motivated by such results or the results of conventional studies can be confirmed by research based on reused routine data [14].

### 4.3. Statistical Analysis

We defined wide inclusion criteria for the HiGHmed patient cohort (see above). In summary, we could compute MAGGIC scores for 894 patients and BioHFv1 scores for 1353 complete records + 560 imputed records = 1913 patients. The origins of incomplete/missing documentation differed widely across partner sites and are best explained by site-specific data acquisition procedures. We soon realized that score distributions significantly differed across partner sites as well and identified a number of reasons in the respective patient subpopulations at each partner site: proportion of in- vs. outpatients, medication (beta blockers and ACE inhibitors), and LVEF (see Figure 3 and Figure 4 for details).

Generally, we cannot recommend the imputation of BioHFv1 heart failure scores in general. Score distributions based on imputed values were shifted to higher median values in comparison to scores computed from complete data records. This was observed for all sites except for Würzburg.

In future analyses, we plan to investigate whether the computed HF scores match the observed outcome (mortality) and whether machine learning algorithms could perform better on the same data, in terms of risk predictions.

## Figures and Tables

**Table 1:** Data items from the openEHR templates were used to calculate the MAGGIC and Barcelona Heart Failure Score (LOINC = Logical Observation Identifiers Names and Codes, a standard for identifying medical laboratory observations).

OpenEHR Template	Data Items *
Personal Data	Year of birth (B, M)
Patient history	Sex (B, M) Weight (M) Height (M) Systolic blood pressure (M) NYHA dysfunctional class (B, M) Current smoking (M) Time of first diagnosis of HF (M) Diabetes mellitus (M) Chronic lung disease (M)
Medication	Betablocker (B, M) ACEi/ARB (B, M) Statin (B) Loop diuretic (B)
Echocardiography	LV ejection fraction (B, M)
Laboratory Data	Creatinine (M) [LOINC 14682-9, 2160-0] Sodium (B) [LOINC 2951-2] Hemoglobin (B) [LOINC 718-7, 30350-3] Estimated GFR (B) [LOINC 62238-1]
-	Inpatient/Outpatient

* M = required for MAGGIC Score; B = required for Barcelona Score.

**Table 2:** The primary systems used for data integration in the UCC for all participating sites.

Template	Hannover	Heidelberg	Göttingen	Würzburg	Berlin
Personal Data	SAP i.s.h.med	SAP i.s.h.med	SAP IS-H	SAP i.s.h.med	SAP IS-H, Custom ^1^
Patient History	Custom ^1^	Custom ^1^	Custom ^1^	SAP i.s.h.med, ignimed ^1^	Custom ^1^
Medication	Custom ^1^	Custom ^1^	Custom ^1^	SAP i.s.h.med, Meona	Custom ^1^
Echocardiography	Philips IntelliSpace	MySQL DB solution	GE Healthcare Carddas	KardioText EchoText	Custom ^1^
Laboratory Data	OSM Opus::L	Nexus Swisslab	OSM Opus::L	Nexus Swisslab	Medat; GLIMS

^1^ Custom forms were created for the UCC which are filled out by study nurses.

**Table 3:** Upper and lower limits of continuous variables for plausibility checks before score calculation. * HF duration: heart failure duration.

Variable	Lower Limit	Upper Limit	Unit
Age	18	110	years
BMI	14	60	kg/m^2^
Blood pressure systolic	70	250	mm [Hg]
HF duration *	0	Age * 12	months
LV-EF	4	85	%
Creatinine	26.526	1326.3	µmol/L
Sodium	120	150	mmol/L
Hemoglobin	5	20	g/dL
Estimated GFR	5	120	mL/min/1.73

**Table 4:** Number of patients and MAGGIC Score calculations for all partner sites.

	Partner Sites				
Patient Type	Berlin *	Göttingen	Hannover	Heidelberg	Würzburg
Inpatient	0	26	353	0	14
Outpatient	0	77	15	156	253
Total sum	0	103	368	156	267

* Berlin site excluded from MAGGIC analysis.

**Table 5:** Number of patients and BioHFv1 score calculations for all partner sites.

	Partner Sites (Complete/Imputed)				
Patient Type	Berlin *	Göttingen	Hannover	Heidelberg	Würzburg
Inpatient	20/38	35/45	701/172	0	14/4
Outpatient	(1)	105/148	22/3	179/85	277/52
Total sum	20/38	139/193	723/175	179/85	291/56

* We excluded the single Berlin outpatient in subsequent analyses.

## Data Availability

Data will be made available upon publication.

## Appendix A. AQL Queries

**Query A1.** AQL query executed to retrieve data items from the study participation template.

SELECT distinct e/ehr_status/subject/external_ref/id/value as id,

 e/ehr_id/value as ehr_id,

 x/items[at0002]/value/value as studie

FROM EHR e

CONTAINS COMPOSITION c

CONTAINS CLUSTER x[openEHR-EHR-CLUSTER.study_details.v1]

WHERE x/items[at0002]/value/value = ‘HiGHmed-UCC’

**Query A2.** AQL query executed to retrieve data items from the personal details template.

SELECT distinct e/ehr_status/subject/external_ref/id/value as id,

 b/items[at0001]/value/value as birthDate

FROM EHR e

CONTAINS COMPOSITION c

CONTAINS CLUSTER b[openEHR-EHR-CLUSTER.person_birth_data_iso.v0]

**Query A3.** AQL query executed to retrieve data items from the medical history template.

SELECT distinct e/ehr_status/subject/external_ref/id/value as id,

 c/context/start_time/value as time,

 g/data[at0002]/items[at0022]/value/value as gender,

 w/data[at0002]/events[at0003]/data[at0001]/items[at0004]/value/magnitude as weight_m,

 w/data[at0002]/events[at0003]/data[at0001]/items[at0004]/value/units as weight_u,

 h/data[at0001]/events[at0002]/data[at0003]/items[at0004]/value/magnitude as height_m,

 h/data[at0001]/events[at0002]/data[at0003]/items[at0004]/value/units as height_u,

 s/data[at0001]/events[at0006]/data[at0003]/items[at0004]/value/magnitude as sys_bp_m,

 s/data[at0001]/events[at0006]/data[at0003]/items[at0004]/value/units as sys_bp_u,

 n/data[at0001]/events[at0002]/data[at0003]/items[at0004]/value/value as nyha,

 sm/data[at0001]/items[at0089]/value/value as smoking,

 hf/data[at0001]/items[at0077,’Erstdiagnose Herzinsuffizienz’]/value/value as hf_diagnosis_date

FROM EHR e

 CONTAINS COMPOSITION c

 CONTAINS (EVALUATION g[openEHR-EHR-EVALUATION.gender.v1] or

 OBSERVATION w[openEHR-EHR-OBSERVATION.body_weight.v2] or

 OBSERVATION h[openEHR-EHR-OBSERVATION.height.v2] or

 OBSERVATION s[openEHR-EHR-OBSERVATION.blood_pressure.v2] or

 OBSERVATION n[openEHR-EHR-OBSERVATION.nyha_heart_failure.v1] or

 EVALUATION sm[openEHR-EHR-EVALUATION.tobacco_smoking_summary.v1] or

 EVALUATION hf[openEHR-EHR-EVALUATION.problem_diagnosis.v1, ‘Erstdiagnose Herzinsuffizienz’]

)

WHERE c/archetype_details/template_id/value = ‘Anamnese’

**Query A4.** AQL query executed to retrieve diagnosis data items from the medical history template.

SELECT distinct e/ehr_status/subject/external_ref/id/value as id,

 c/context/start_time/value as time,

 d1/data[at0001]/items[at0002]/value/value as diabetes_yes,

 d2/data[at0001]/items[at0003,’Problem/Diagnose’]/value/value as diabetes_no,

 co1/data[at0001]/items[at0002]/value/value as copd_yes,

 co2/data[at0001]/items[at0003,’Problem/Diagnose’]/value/value as copd_no

FROM EHR e

CONTAINS COMPOSITION c

 CONTAINS (

 SECTION s1[openEHR-EHR-SECTION.adhoc.v1, ‘Diabetes Mellitus’]

  CONTAINS (EVALUATION d1[openEHR-EHR-EVALUATION.problem_diagnosis.v1] or EVALUATION d2[openEHR-EHR-EVALUATION.exclusion_specific.v1]) or

 SECTION s2[openEHR-EHR-SECTION.adhoc.v1, ‘Chronische Lungenerkrankung’]

  CONTAINS (EVALUATION co1[openEHR-EHR-EVALUATION.problem_diagnosis.v1] or EVALUATION co2[openEHR-EHR-EVALUATION.exclusion_specific.v1])

)

**Query A5.** AQL query executed to retrieve data items from the medication template.

SELECT distinct e/ehr_status/subject/external_ref/id/value as id,

 c/context/start_time/value as time_med,

 m/items[at0001,’Arzneimittel-Code’]/value/defining_code/code_string as med_code

FROM EHR e

CONTAINS COMPOSITION c

CONTAINS (

 OBSERVATION x[openEHR-EHR-OBSERVATION.medication_statement.v0] and

 CLUSTER m[openEHR-EHR-CLUSTER.drug_class.v0])

WHERE m/items[at0001,’Arzneimittel-Code’]/value/defining_code/code_string MATCHES {‘ACEHem’, ‘AT1RANT’, ‘BETABL’, ‘STAT’, ‘ALDANT’, ‘SCHDIU’, ‘VALS+SACU’} and

 x/protocol[at0004]/items[openEHR-EHR-CLUSTER.medication_status_fhir.v0]/items[at0001]/value/value = ‘Aktiv’

**Query A6.** AQL query executed to retrieve data items from the echocardiography template.

SELECT distinct e/ehr_status/subject/external_ref/id/value as id,

 l/items[at0001]/items[at0002]/value/magnitude as lvef_m,

 l/items[at0001]/items[at0002]/value/units as lvef_u,

 c2/context/start_time/value as time

FROM EHR e

CONTAINS COMPOSITION c2

CONTAINS CLUSTER l[openEHR-EHR-CLUSTER.imaging_result.v0, ‘Linksventrikuläre Auswurffraktion (LV-EF)’]

**Query A7.** AQL query executed to retrieve data items from the laboratory data template.

SELECT distinct e/ehr_status/subject/external_ref/id/value as id,

   l/items[at0024]/value/value as analyt,

   l/items[at0024]/value/defining_code/terminology_id/value as terminology,

   l/items[at0024]/value/defining_code/code_string as code,

   l/items[at0001]/value/magnitude as magnitude,

   l/items[at0001]/value/units as unit,

 c/context/start_time/value as time

FROM EHR e

CONTAINS COMPOSITION c

CONTAINS OBSERVATION o[openEHR-EHR-OBSERVATION.laboratory_test_result.v1]

CONTAINS CLUSTER l[openEHR-EHR-CLUSTER.laboratory_test_analyte.v1]

WHERE l/items[at0024]/value/defining_code/terminology_id/value = ‘LOINC’ and

 l/items[at0024]/value/defining_code/code_string MATCHES {‘14682-9’,‘2160-0’,‘2951-2’,‘718-7’,‘30350-3’,‘33762-6’,‘83107-3’,‘62238-1’,‘67151-1’}
